# Data of de novo transcriptome assembly of the myxozoan parasite *Tetracapsuloides bryosalmonae*

**DOI:** 10.1016/j.dib.2021.106831

**Published:** 2021-02-04

**Authors:** Gokhlesh Kumar, Reinhard Ertl, Frank Nilsen, Jerri L. Bartholomew, Mansour El-Matbouli

**Affiliations:** a Clinical Division of Fish Medicine, University of Veterinary Medicine Vienna, Vienna, Austria; b VetCore Facility, University of Veterinary Medicine Vienna, Vienna, Austria; c Sea Lice Research Centre, Department of Biological Sciences, University of Bergen, Bergen, Norway; d Department of Microbiology, Oregon State University, Corvallis, United States of America

**Keywords:** Malacosporean, Parasite sacs, RNA-seq, Bryozoan, Proliferative kidney disease, Salmonids

## Abstract

*Tetracapsuloides bryosalmonae,* a myxozoan endoparasite, causes proliferative kidney disease in salmonids. The life cycle of *T. bryosalmonae* occurs between invertebrate bryozoan and vertebrate fish hosts. *T. bryosalmonae* develops in the body cavity of colonial bryozoan and spores are released from mature spore sacs into the water likely through the vestibular pore and infect fish by attaching to their gills. However, very little is known about the transcriptome of this important parasite, which hampers studies into the molecular mechanisms of host-parasite interactions and understanding the parasite biology. In order to circumvent this limitation, we performed *de novo* transcriptome assembly on the sacs of *T. bryosalmonae*, collected from infected bryozoan *Fredericella sultana*. A total of 111.5 million filtered paired-end reads was obtained and assembled into 25,908 contigs corresponding to putative transcripts that were functionally annotated. More than 50% of the assembled transcripts (13,071 contigs) had a significant hit in NCBI non-redundant database. Based on Gene ontology annotation, the most highly scored categories of molecular function of the contigs were related to binding and catalytic activities in *T. bryosalmonae*. This study provides a global overview of the *T. bryosalmonae* transcriptome that will be a valuable resource for identifying virulence factors, gene discovery, genome annotation, and vaccine development applications. This data is accessible via NCBI BioProject (PRJNA680464).

## Specifications Table

**Table:** 

Subject	Parasitology
Specific subject area	Transcriptomics
Type of data	Assembly, Table, Figure
How data were acquired	Illumina NextSeq 550
Data format	Raw reads (fastq), Assembly (fasta)
Parameters for data collection	*Tetracapsuloides bryosalmonae* sacs were collected from infected *Fredericella sultana* and were used for library preparation and sequencing.
Description of data collection	Total RNA was extracted from 6 parasite sac samples using QIAgen RNeasy mini kit and included an on-column DNase digestion step. cDNA library were prepared with the TruSeq RNA Sample Prep Kit and high-throughput sequencing on an Illumina NextSeq 550 platform using 150-bp paired-end reads.
Data source location	Parasite sacs used in this experiment originated from laboratory infected bryozoan *Fredericella sultana*, University of Veterinary Medicine Vienna
Data accessibility	Raw data and final assembled contigs were deposited in the NCBI database under the Bioproject accession number PRJNA680464 https://www.ncbi.nlm.nih.gov/bioproject/PRJNA680464 The final transcriptome assembly has been deposited in Figshare 10.6084/m9.figshare.13302746https://figshare.com/s/d1fea33991155e5da7b0 The associated annotation data are available as Supplementary Material.

## Value of the Data

• We report Illumina sequencing and *de novo* assembly of the transcriptome from the sacs of *T. bryosalmonae*. It is a basal myxozoan and the data will be important for understanding of parasite biology and the evolution of this taxa by phylogenetic studies*.*
• The data will facilitate genome annotation and discovery of novel gene candidates that could be used for disease prevention strategies of *T. bryosalmonae*, for example, by silencing of important virulence genes, such as protease and motility genes.
• The assembled coding sequences of transcripts associated with numerous biological, molecular function, and cellular processes can be used for designing primers and probes for gene expression studies to evaluate metabolic and virulence factors of *T. bryosalmonae* in the infected hosts.

## 1 Data Description

**Fig. 1:**
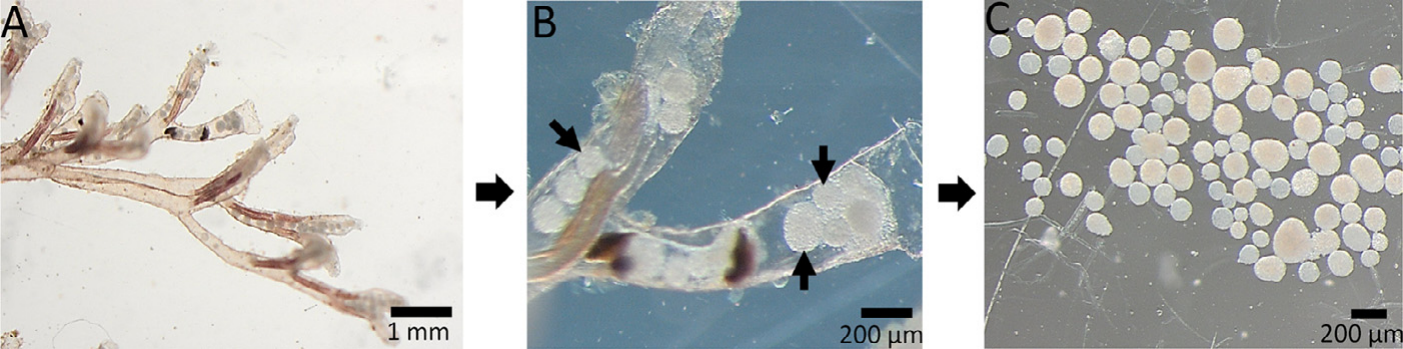
Flowchart of the experimental setup used to collect parasite sacs from infected bryozoa for sequencing. (A) Laboratory infected bryozoan *Fredericella sultana* colony. (B) Infected zooid containing the parasite sacs (arrows). *F. sultana* zooids from different colonies were used for the collection of parasite sacs. (C) Isolated pure parasite sacs. RNA was isolated from 6 parasite sacs samples. In a total ∼111.5 million clean paired-end reads were obtained. *De novo* assembly of filtered reads was performed with CLC Genomic Workbench 12 software and was annotated using Blast2GO.

In this omics era, knowledge about proliferative kidney disease has improved significantly, yet the fundamental underlying virulence mechanisms of *T. bryosalmonae* are still poorly understood. There is no reference genome publicly available to date. Identification of key virulence factors would support management of a disease that has been difficult to control in both aquaculture and wild fisheries. We generated first-time Illumina sequencing and *de novo* assembly of the transcriptome from the sacs of *T. bryosalmonae*. A description of collection of the parasite sacs from infected bryozoan colonies for sequencing is presented in a flowchart (Fig. 1). Paired-end sequences obtained from 6 parasite sacs samples were assembled with CLC Genomic Workbench software (Qiagen, Denmark) to generate the *T. bryosalmonae* transcriptome. The clustering tool cd-hit-est was used to remove redundant sequences. Functional annotations were added with Blast2GO software, based on similarity BLASTX analysis against NCBI's non-redundant protein database.

### 1.1 RNA-seq data quality

The demultiplexing of the raw reads was achieved based on unique barcodes introduced during library preparation. The quality of the sequencing reads from the Illumina platform was evaluated using FastQC. The quality scores of the reads were stable across the reads and 92% of the called bases had a Phred score ≥ 30, indicating the base quality is highly sufficient. The median base quality of the ends of the reads, which are often of lower quality, was still within an acceptable range (Supplementary Fig. 1A). Additionally, histograms of the numbers of reads against the average read quality were produced (Supplementary Fig. 1B) and showed that the quality of most of the reads was around Q34. A total of 234 million reads were generated (117,478,076 paired-end reads), with an average of 39 million reads per sample. Approximately 222 million reads (94.92%) were retained after removing the adapters and trimming for quality. These results indicated that the sequence quality was sufficient for downstream analyses.

### 1.2 Transcriptome assembly

**Fig. 2:**
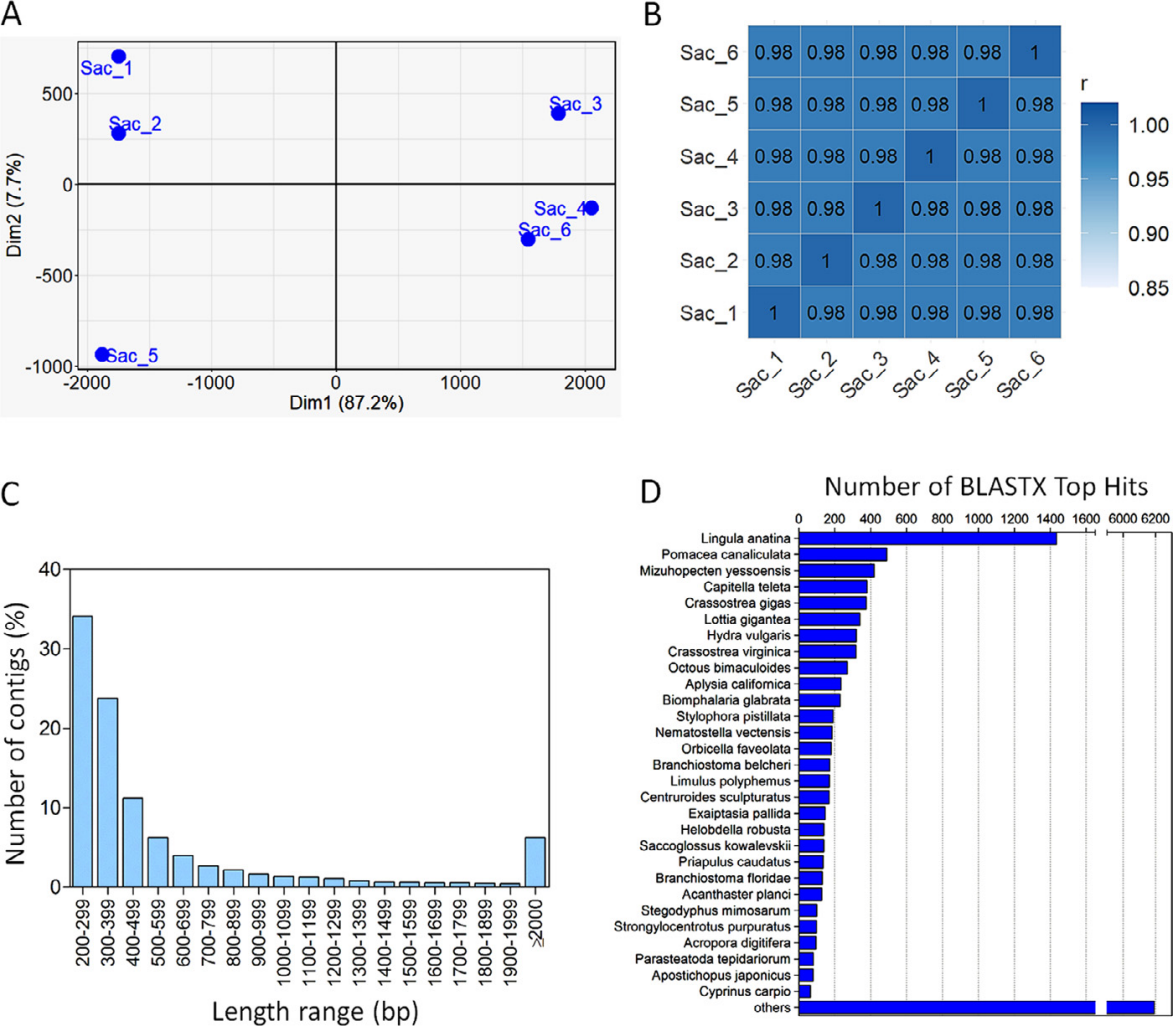
Quality control of the transcriptome data. (A) PCA plot of normalized RNA-seq expression values of six samples. PCA analysis based on the normalized TPM expression values showed a high degree of uniformity between the replicates. (B) Heatmap showing Pearson's correlation coefficient (r) for TPM normalized values across samples, indicating positive correlation between biological replicates. (C) Sequence length distribution (bp) of contigs assembled from Illumina RNA-seq reads. The y-axis represents the percentage of contigs assigned to each length range, (D) Species distribution of BLASTX hits. Number of hits for the most represented species from BLASTX analysis of *Tetracapsuloides bryosalmonae* transcripts against the non-redundant protein database. The species with the most significant hit for each transcript was taken into account.

The removal of redundant sequences of samples resulted in a total of 25,908 contigs with an average length of 687 bp and 37.9% GC content. A summary of the sequencing and assembly statistics of the sacs is presented in Table 1. An overview of annotated contigs with all the details is presented in Supplementary File 1. Quantitative expression levels of *T. bryosalmonae* transcripts sorted by TPM are presented in Supplementary File 2. PCA analysis based on the normalized expression values demonstrated a high degree of uniformity between the replicates (Fig. 2A) and showed positive correlation between them (Fig. 2B), indicating an overall highly uniform gene expression status between replicates and reproducible sample pre-processing. The majority of contigs was distributed within a size range of 200 bp to 299 bp and 6.2% of the contigs were larger than 2000 bp. The lengths of the assembled contigs are represented as a bar chart (Fig. 2C). The numbers of reads mapped back to contigs resulted in > 95% mapped reads. BLASTX top hits retrieved from the NCBI database represented only 29 top hits with similarity to *T. bryosalmonae*. The largest number of blast hits to a single species (>1400) for the *T. bryosalmonae* transcripts were derived from the brachiopod species *Lingula anatina*, followed by *Pomacea canaliculata* and *Mizuhopecten yessoensis* (Fig. 2D). More than 6000 transcripts represented similarities with other species.

**Table 1:** Summary statistics of *de novo* transcriptome assembly for *Tetracapsuloides bryosalmonae* sacs using the combined data of 6 samples. Transcriptome assembly statistics were generated using CLC Genomics Workbench 12 software. Reads used for the *de novo* assembly were trimmed for Illumina adapters and quality filtered.

Transcriptome features	Values
Number of raw reads (paired)	117,478,076
Number of filtered reads (paired)	111,517,126
Number of contigs	26,207
Number of contigs after redundancy filtration	25,908
Average contig length (bp)	687
N50 (bp)	1079
GC-content	37.9%
Percentage of reads mapped back to transcripts	95.2%
Contigs with BLASTX hits	13,071
Annotated contigs	11,291

**Fig. 3:**
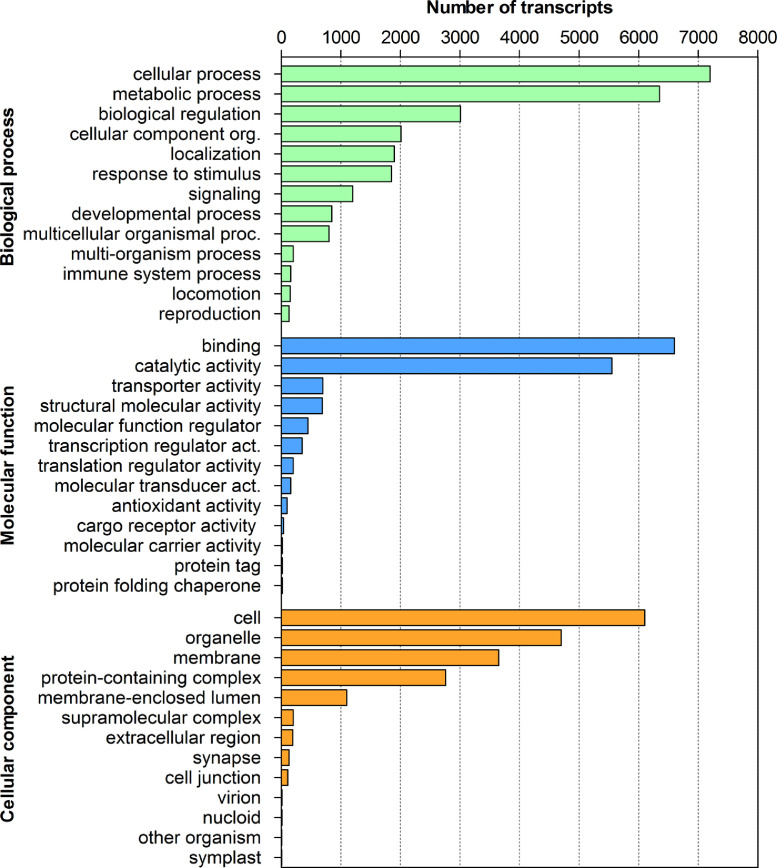
Gene ontology annotation of *Tetracapsuloides bryosalmonae* transcripts. Most frequent level 2 GO terms in *T. bryosalmonae* transcripts, separated for the GO domains, biological process, molecular function, and cellular component.

Based on GO annotation, transcripts were assigned to GO terms categorized into three GO domains: biological process (58,025), molecular function (32,545), and cellular component (38,773) (Fig. 3). Classification of the GO terms showed that within the biological processes the most common were metabolic and cellular processes that were represented by more than 6000 transcripts. Biological regulation was the third most common process represented by approximately 3000 transcripts. More than 6000 transcripts were related with binding activity in the molecular functions and in the cellular components.

### 1.3 Data records

The total raw sequencing data from 6 parasite sacs samples was used for assembly in the present study. Data generated in this study have been deposited in the NCBI/GenBank database with the Bioproject accession number PRJNA680464. The raw reads have been deposited in the NCBI Sequence Read Archive database under the accession numbers: SRR13124483, SRR13124484, SRR13124485, SRR13124486, SRR13124487, and SRR13124488. The final transcriptome assembly has been deposited in the NCBI Transcriptome Shotgut Assembly database under the accession number GIXG00000000 and in Figshare under the DOI: 10.6084/m9.figshare.13302746.

## 2 Experimental Design, Materials and Methods

### 2.1 Sample preparation

Colonies of the bryozoan, *Fredericella sultana* were grown in controlled laboratory conditions. The colonies were cohabitated with *T. bryosalmonae*-infected brown trout for 2 weeks. After cohabitation, infected colonies were fed algae species (*Cryptomonas ovata, Cryptomonas species*, and *Synechococcus species*) and maintained under optimal laboratory conditions according to Kumar et al. [1]. After visualizing the parasite sacs under dissecting microscope, infected zooids (Fig. 1B) were dissected with fine needles and forceps using a dissecting microscope (Olympus SZX10, Japan). The clean and pure parasite sacs (Fig. 1C) were collected from different infected zooids by sterile pipette and transferred into 2 ml microcentrifuge tubes to obtain six independent biological replicates, preserved in RNA later and stored at −80 °C.

### 2.2 Library preparation

Total RNA was isolated from the sacs using the RNeasy Mini Kit and included an on-column DNase digestion step according to the manufacturer's protocol (Qiagen, Germany). The integrity of the RNA was measured on the 4200 TapeStation using the RNA ScreenTape assay (Agilent Technologies, USA). RNA samples with an RNA integrity number above 8.0 were used for library preparation. A total of 6 cDNA libraries were prepared out of 300 ng total RNA input with the TruSeq RNA Sample Prep Kit v2 (Illumina, USA) according to the manufacturer's protocol. Library quality control was assessed on the 4200 TapeStation with the D1000 ScreenTape Kit (Agilent Technologies). Libraries were pooled and sequenced on one lane of an Illumina NextSeq 550 sequencing instrument using 150-bp paired-end reads. Sequencing was done by the Vienna BioCenter Core Facilities NGS unit (Vienna, Austria).

### 2.3 Transcriptome assembly

Illumina sequencing generated 117,478,076 paired-end reads from the pooled cDNA libraries (Table 1). Sequence filtering was performed in CLC Genomics Workbench 12 software (Qiagen, Denmark). Adapter sequences, low quality reads (Phred score ≤ 30) and reads shorter than 50 bp were removed. The resulting filtered reads were assembled using the *de Bruijn graph*-based *de novo* assembler of CLC Genomics [2]. Assembly parameters: *k-mer* and bubble size were varied to optimize the assembled contigs. The final assembly (minimum contig length = 200 bp) was done with *k-mer* = 35, and bubble size = 300, which was based on the output parameters: high N50, low total number of contigs, high average contig length and high percentage of reads mapped back to transcripts. The cluster tool cd-hit-est with a sequence identity threshold of 0.95 was used for redundancy filtration of the assembly [3]. Numbers of reads mapping back to the contigs were converted to transcripts per million (TPM) expression values [4] to estimate the transcript abundance. In the initial data investigation, we also performed a principal component analysis and global Pearson correlation analysis to test the significance of the clusters and correlation between samples.

### 2.4 Functional annotation and gene ontology

To assign the putative functions to the non-redundant transcripts of *T. bryosalmonae* (*n* = 25,908), similarity search using BLASTX was performed with the software Blast2GO [5] against the NCBI non-redundant protein database [6] with an *E*-value cut-off of ≤ 1.0E-3. To monitor for *F. sultana* and algae contamination, we mapped assembled contigs against the *F. sultana* transcriptome assembly (NCBI TSA accession: GHLZ01000000) [7] and algae genomes (*Synechococcus* spp. and *Cryptomonas* spp*.*). Based on these results, gene ontology (GO) terms of the three GO domains: Biological Process, Molecular Function and Cellular Component were separately assigned to the contigs in Blast2GO. The ‘GO slim’ function in Blast2GO was used to merge specific GO terms into higher-order terms for each contig in order to provide a more general overview of the GO term distribution over the whole transcriptome.

## Ethics Statement

The cohabitation of bryozoan *F. sultana* colonies with infected brown trout was conducted in accordance with the relevant guidelines and regulations §26 of the Austrian Law for Animal Experiments, Tierversuchsgesetz 2012. The institutional ethics committee of the University of Veterinary Medicine, Vienna, Austria and the national authority approved this experiment under the permission numbers BMWFW GZ: 68.205/0181-WF/V/3b/2017 and BMWFW GZ: 2020–0.237.729.

## Funding Information

This study was funded by the Austrian Science Fund (FWF) project no. P 30981-B32 to GK.

## CRediT Author Statement

**Gokhlesh Kumar, Frank Nilsen, Jerri L. Bartholomew, Mansour El-Matbouli:** Conceptualization, Methodology; **Gokhlesh Kumar:** Funding acquisition, Investigation, Validation, Writing Original draft; **Reinhard Ertl:** Formal analysis, Validation, Data Curation; **Frank Nilsen, Jerri L. Bartholomew, Mansour El-Matbouli:** Reviewing and Editing.

## Declaration of Competing Interest

The authors declare that they have no known competing financial interests or personal relationships which have or could be perceived to have influenced the work reported in this article.

## Appendix Supplementary materials

**Supplementary File** 1: List of *Tetracapsuloides bryosalmonae* transcripts. This file contains all non-redundant *T. bryosalmonae* contigs including functional annotation and gene ontology data. Image, application 1

**Supplementary File 2**: Quantitative expression levels of *Tetracapsuloides bryosalmonae* transcripts. List of all contigs sorted by TPM (transcripts per million) expression values. Image, application 2

**Supplementary Figure 1:** Quality control of the transcriptome data. (A) Base quality using Phred scores for the data from Illumina NextSeq 550. In the box plots, the whiskers represent the range between the 10 and 90% quantiles; the yellow boxes represent the range between the upper and lower quartiles; the red lines represent median. The blue line indicates the mean quality, (B) Distribution of read-over-read quality. Image, application 3

## Footnotes

Supplementary material associated with this article can be found in the online version at doi:10.1016/j.dib.2021.106831.
