# Supplementary figures and images for: Data of de novo transcriptome assembly of the myxozoan parasite Tetracapsuloides bryosalmonae
Source: Data Brief. 2021 Feb 4;35:106831. doi: 10.1016/j.dib.2021.106831 (PMC7890133; doi:10.1016/j.dib.2021.106831)

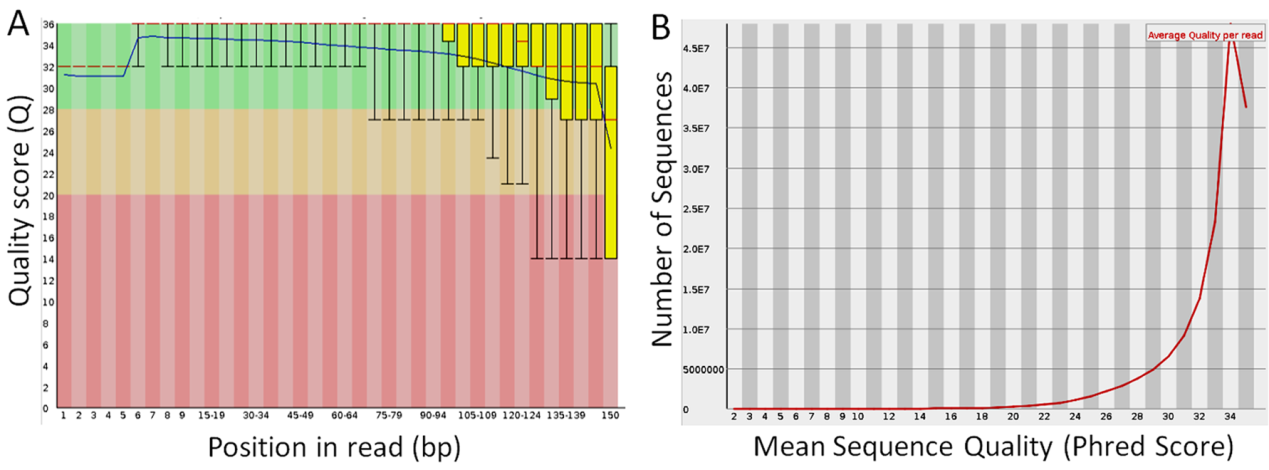

Supplement: Supplementary file 3 — Supplementary Figure 1: Quality control of the transcriptome data. (A) Base quality using Phred scores for the data from Illumina NextSeq 550. In the box plots, the whiskers represent the range between the 10 and 90% quantiles; the yellow boxes represent the range between the upper and lower quartiles; the red lines represent median. The blue line indicates the mean quality, (B) Distribution of read-over-read quality. [file mmc3.docx]
